# Association between *Thiopurine S-methyltransferase* Polymorphisms and Thiopurine-Induced Adverse Drug Reactions in Patients with Inflammatory Bowel Disease: A Meta-Analysis

**DOI:** 10.1371/journal.pone.0121745

**Published:** 2015-03-23

**Authors:** Yue-Ping Liu, Hai-Yan Wu, Xiang Yang, Han-Qing Xu, Yong-Chuan Li, Da-Chuan Shi, Jun-Fu Huang, Qing Huang, Wei-Ling Fu

**Affiliations:** Department of Laboratory Medicine, Southwest Hospital, Third Military Medical University, Chongqing, 400038, China; Harvard Medical School, UNITED STATES

## Abstract

**Purpose:**

Thiopurine drugs are well established treatments in the management of inflammatory bowel disease (IBD), but their use is limited by significant adverse drug reactions (ADRs). Thiopurine S-methyltransferase (*TPMT*) is an important enzyme involved in thiopurine metabolism. Several clinical guidelines recommend determining *TPMT* genotype or phenotype before initiating thiopurine therapy. Although several studies have investigated the association between *TPMT* polymorphisms and thiopurine-induced ADRs, the results are inconsistent. The purpose of this study is to evaluate whether there is an association between *TPMT* polymorphisms and thiopurine-induced ADRs using meta-analysis.

**Methods:**

We explored PubMed, Web of Science and Embase for articles on *TPMT* polymorphisms and thiopurine-induced ADRs. Studies that compared *TPMT* polymorphisms with-ADRs and without-ADRs in IBD patients were included. Relevant outcome data from all the included articles were extracted and the pooled odds ratio (OR) with corresponding 95% confidence intervals were calculated using Revman 5.3 software.

**Results:**

Fourteen published studies, with a total of 2,206 IBD patients, which investigated associations between *TPMT* polymorphisms and thiopurine-induced ADRs were included this meta-analysis. Our meta-analysis demonstrated that *TPMT* polymorphisms were significantly associated with thiopurine-induced overall ADRs and bone marrow toxicity; pooled ORs were 3.36 (95%CI: 1.82–6.19) and 6.67 (95%CI: 3.88–11.47), respectively. *TPMT* polymorphisms were not associated with the development of other ADRs including hepatotoxicity, pancreatitis, gastric intolerance, flu-like symptoms and skin reactions; the corresponding pooled ORs were 1.27 (95%CI: 0.60–2.71), 0.97 (95%CI: 0.38–2.48), 1.82 (95%CI: 0.93–3.53), 1.28 (95%CI: 0.47–3.46) and 2.32 (95%CI: 0.86–6.25), respectively.

**Conclusions:**

Our meta-analysis demonstrated an association of *TPMT* polymorphisms with overall thiopurine-induced ADRs and bone marrow toxicity, but not with hepatotoxicity, pancreatitis, flu-like symptoms, gastric intolerance and skin reactions. These findings suggest that pretesting the *TPMT* genotype could be helpful in clinical practice before initiating thiopurine therapy. However, white blood cell count analysis should be the mainstay for follow-up.

## Introduction

Inflammatory bowel disease (IBD), with its two major clinical subtypes, Crohn’s disease (CD) and ulcerative colitis (UC), is a polygenic disease that manifests due to environmental trigger factors on the background of a complex genetic predisposition [1, 2]. The thiopurine drug, 6-mercaptopurine (6-MP), and its pro-drug, azathioprine (AZA), have proven to remain the standard of care for both steroid-dependent and chronically active, or steroid-resistant IBD [3, 4]. However, concerns regarding adverse drug reactions (ADRs) have limited the use of these agents as first line medical therapy. In clinical trials, approximately 20% of IBD patients discontinue thiopurine treatment due to adverse events [5]. Bone marrow toxicity (BMT), hepatotoxicity, pancreatitis, gastric intolerance, skin reactions and flu-like symptoms are among the most common reasons to discontinue thiopurine therapy [6].

Thiopurine S-methyltransferase (TPMT) is an important cytoplasmic enzyme catalyzing the methylation of 6-MP, competing with xanthine oxidase (XO) and hypoxanthine guanine phosphoribosyl transferase (HGPRT) to determine the amount of 6-MP metabolized to cytotoxic 6-thioguanine nucleotides (6-TGNs) [7]. The gene encoding for *TPMT* is subject to genetic polymorphisms that have been studied extensively. Approximately 4%–11% of individuals are heterozygous for a mutant *TPMT* allele and have intermediate *TPMT* activity; whereas approximately 1 in 300 individuals are homozygous or compound heterozygous and have very low or absent *TPMT* activity [8–10]. Very low or deficient enzyme activity resulting from polymorphisms in the *TPMT* encoding genes may be associated with thiopurine-induced adverse drug reactions [11]. Several clinical guidelines recommend determining *TPMT* genotype or phenotype before commencing thiopurine therapy [12–14]. Drug label modifications for AZA approved by the U.S. Food and Drug Administration (FDA) also recommend pretesting, but does not mandate it [15]. The evidence base for these recommendations is unclear, particularly the crucial, direct evidence that pre-therapy *TPMT* measuring decreases BMT-specific mortality [16]. In addition, whether there is an association between *TPMT* polymorphisms and thiopurine-induced ADRs is still controversial. For instance, in a study of 219 IBD patients, *TPMT* polymorphisms were significantly associated with pancreatitis, but were not associated with bone marrow toxicity [17]. However, these results were contradicted by another study of 93 IBD patients [18].

At the time of planning our meta-analysis, we identified that a similar meta-analysis of the association of *TPMT* polymorphisms on thiopurine-induced ADRs in thiopurine-treated IBD patients had been undertaken previously in 2010 [19]. However, the previously published study only investigated the association of *TPMT* polymorphisms on thiopurine-induced BMT, hepatotoxicity and pancreatitis, not including all the common thiopurine-induced ADRs. In addition, a large number of *TPMT* pharmacogenetic studies had been published annually since 2010, meaning that our meta-analysis included several more studies investigating *TPMT* polymorphisms. In the present study, we performed a meta-analysis with the purpose of gaining more insight into a possible association between *TPMT* polymorphisms and all the common thiopurine-induced ADRs by evaluation of the literature on this subject. The finding of a significant association may become indirect evidence for pretesting *TPMT* genotype before commencing thiopurine therapy in IBD patients.

## Results

### Literature search outcome

**Fig 1:**
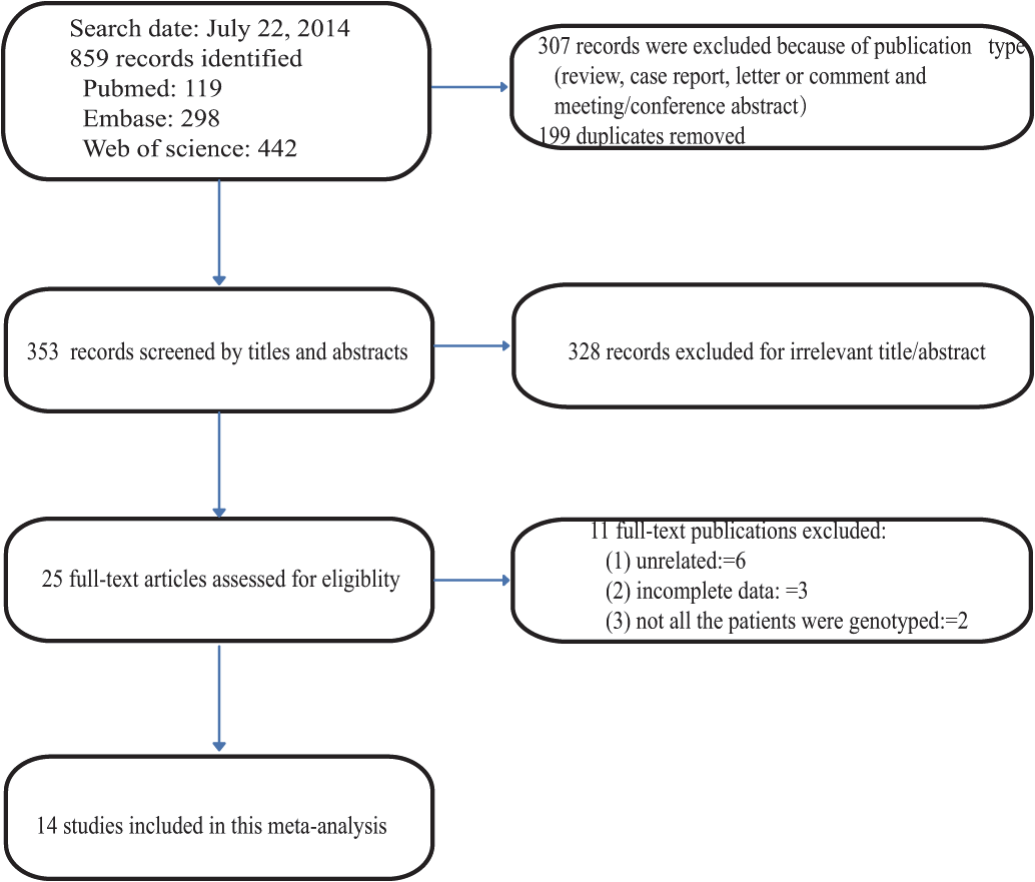
Flowchart describing the systematic literature search and study selection process.

With the aforementioned search strategy, a total of 859 potentially relevant records were retrieved. 307 records were excluded because of publication type (review, case report, letter or comment and meeting/conference abstract). 199 records were excluded because they were duplicates and another 328 records were excluded after reviewing the titles and the abstracts; 25 full-text papers were deemed to be relevant and were examined in detail. 11 full-text papers were excluded for the reasons described in **Fig. 1**(The excluded 11 studies were listed in **S1 Table**). Finally, 14 studies [18, 20–32] met the inclusion criteria, and were included in this meta-analysis.

### Characteristics of included studies

A total of 14 studies with 2,276 IBD patients were included in our meta-analysis and the average number of patients per study was 170, ranging from 25 to 422. A summary of the included studies is listed in **S2 Table**. The earliest study was reported in 2002 [18], while the latest was in 2013 [31]. 12 of the 14 studies were from research in Caucasian populations of European ancestry, while the other two studies were from Asian populations (one from Chinese [30] and the other from Korean [27]). We can see that TPMT*3A is the most common mutant allele in Caucasians while TPMT*3C is the most common in Asians. TPMT*2 was a relatively rare variant allele, which was only found in one study [28]. The frequencies of variant alleles ranged from 0% to 12%. Study types included prospective cohorts and case-sectional cohorts, and only two [20, 26] were prospective cohorts with 79 patients enrolled. As can be observed in **S2 Table**, the definitions of thiopurine-induced BMT markedly varied between studies, but the threshold for the number of leucopenia was generally set at 3–4×10^9^/L, and the number of neutrophils at 1.5×10^9^/L. The definitions of thiopurine-induced hepatotoxicity also differed between studies, with the level of alanine transaminase (ALT) set at >2 times the upper limit of normal (ULN) [18, 21, 24, 26, 31], or at >5 times ULN [30]. Pancreatitis was defined as abdominal pain with elevated amylase or lipase levels, but the elevated levels differed from 2 times the ULN [24, 31] to 4 times the ULN [26]. Gastric intolerance was defined as occurrence of any or a combination of the following: nausea, vomiting, dyspepsia and abdominal pain with normal amylase and normal abdominal ultrasound [26]. 7 [18, 23, 24, 26, 28, 29, 31] of the 14 studies reported the relationship between *TPMT* polymorphisms and thiopurine-induced gastric intolerance. Flu-like symptoms included general malaise, temperature, arthralgia and muscle and joint pains. Skin reactions included rash and allergic reactions.

8 studies determined TPMT*2, *3A, *3B and *3C alleles [20, 21, 23, 27, 28, 30–32], 4 studies determined TPMT*3A, *3B and *3C [24–26, 29]; while TPMT*2, *3A,*3B,*3C,*3D alleles were determined in 1 study [18], and TPMT*2, *3A,*3B,*3C, *3D, *4, *5, *6, *7, *8, *10 alleles were determined in a study by *Hindorf* et al [22]. When all the studies were considered, including a total of 2,276 patients, one compound heterozygous and six homozygous mutant genotypes were detected, with a frequency of approximately 1/325.

### Meta-analysis outcomes

#### TPMT polymorphisms and thiopurine-induced overall ADRs

**Fig 2:**
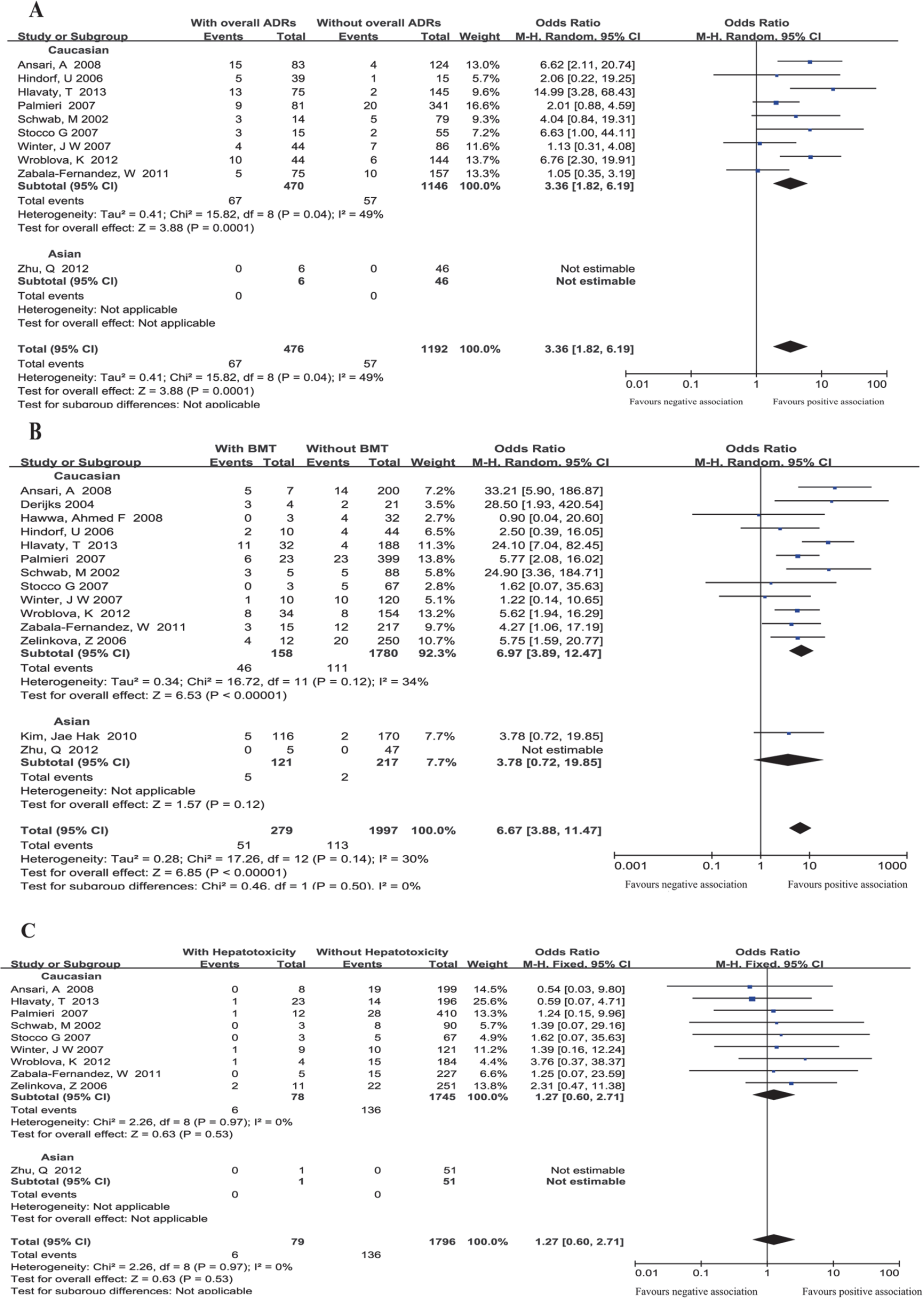
Studies in overall ADRs, BMT and hepatotoxicity subsets were from two ethnic origins, and subgroup analysis was performed in overall ADRs, BMT and hepatotoxicity subsets. Forest plots of association between *TPMT* polymorphisms and thiopurine-induced overall ADRs (A), bone marrow toxicity (B) and hepatotoxicity (C). Total: total number of patients with or without ADRs. Events: number of patients with one or more *TPMT* alleles within the ADRs or no ADRs group.

10 studies [18, 22–24, 26, 28–32], including 1,658 patients, analyzed the association between *TPMT* polymorphisms and overall ADRs. Of the 476 patients with overall ADRs, 67 (14.1%) patients were *TPMT* polymorphism positive and 57 (4.78%) out of 1,192 patients without overall ADRs were *TPMT* polymorphisms positive. The pooled OR (3.36, 95%CI: 1.82–6.19) indicated a significant association between *TPMT* polymorphisms and thiopurine-induced overall ADRs (Fig. 2A).

#### TPMT polymorphisms and thiopurine-induced BMT

All the included studies, with 2,276 patients, reported the association between *TPMT* polymorphisms and BMT. Of 279 patients with BMT, 51 (18.3%) were *TPMT* polymorphisms positive, compared with 113 (5.7%) of the 1,997 patients without BMT. There was a significant association between *TPMT* polymorphisms and BMT (pooled OR = 6.67, 95%CI = 3.88–11.47) (Fig. 2B).

#### TPMT polymorphisms and thiopurine-induced hepatotoxicity

10 studies [18, 21, 23, 24, 26, 28–32] that included 1,875 patients reported the correlation between *TPMT* polymorphisms and hepatotoxicity. Of the 79 patients with hepatotoxicity, 6 (7.6%) were *TPMT* polymorphisms positive, compared with 136 (7.6%) of 1,796 the patients without hepatotoxicity. The overall OR (1.27, 95%CI: 0.60–2.71) demonstrated that *TPMT* polymorphisms did not predict thiopurine-induced hepatotoxicity (Fig. 2C).

#### TPMT polymorphisms and thiopurine-induced pancreatitis

**Fig 3:**
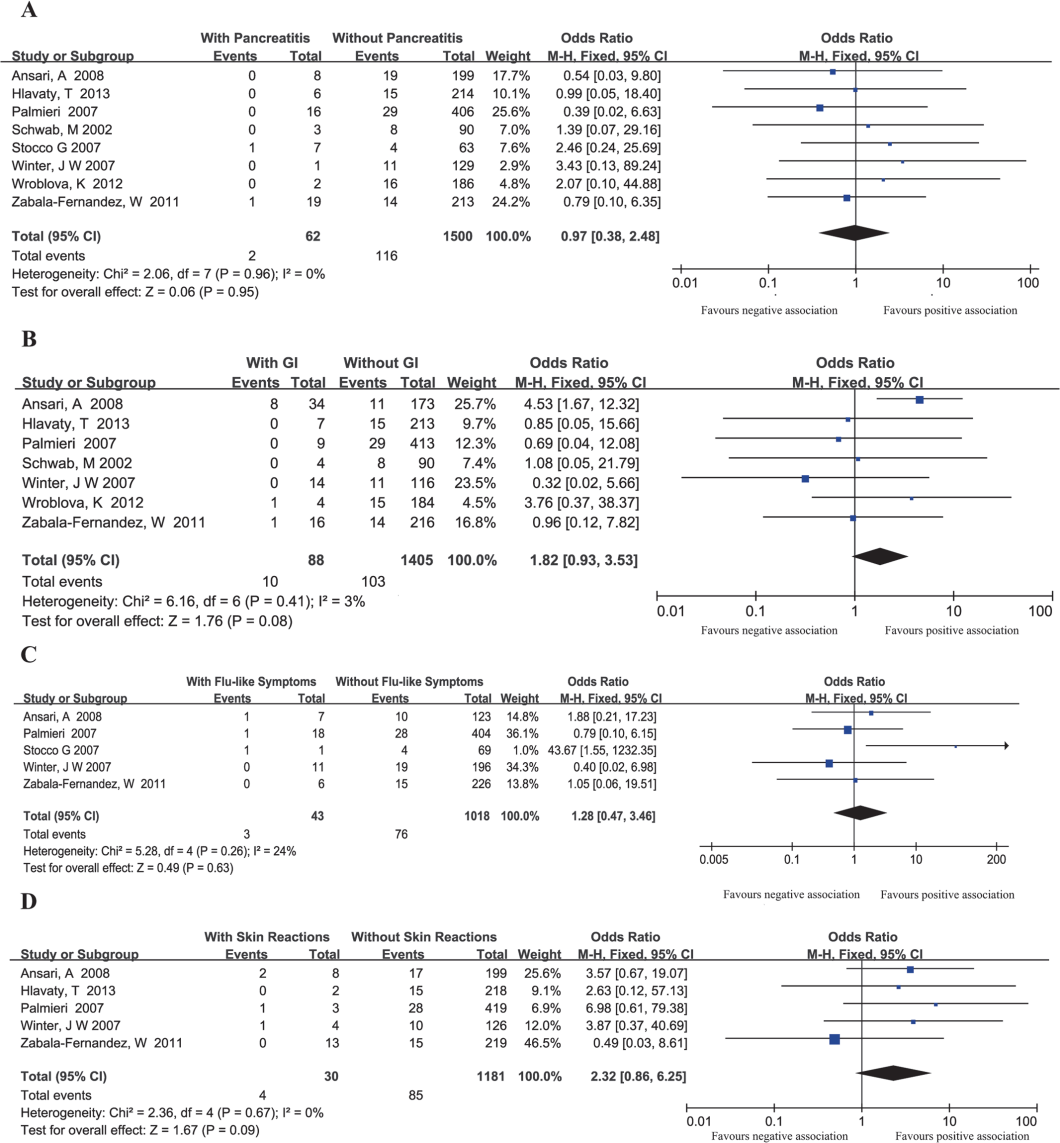
Studies in pancreatitis, gastric intolerance, flu-like symptom and skin reactions subsets were Caucasian population only, and subgroup analysis was not performed in these subsets. Forest plots of association between *TPMT* polymorphisms and thiopurine-induced pancreatitis (A), gastric intolerance (B), flu-like symptoms (C) and skin reactions (D).

8 studies [18, 23, 24, 26, 28, 29, 31, 32] that included 1,562 patients analyzed the association between *TPMT* polymorphisms and pancreatitis. Of the 62 patients with thiopurine-induced pancreatitis, 2 (3.3%) were *TPMT* polymorphisms positive, while 116 (7.7%) of the 1500 patients without pancreatitis were *TPMT* polymorphisms positive. The pooled OR (0.97, 95%CI: 0.38–2.48) indicated that there was no significant difference in *TPMT* polymorphisms in IBD patients with and without thiopurine-induced pancreatitis (Fig. 3A).

#### TPMT polymorphisms and other ADRs (gastric intolerance, flu-like symptoms and skin reactions)

7 studies [18, 23, 24, 26, 28, 29, 31] reported the relationship between *TPMT* polymorphisms and thiopurine-induced gastric intolerance. 5 studies [23, 24, 26, 28, 32] reported flu-like symptoms, while another 5 studies [23, 24, 26, 28, 31] described skin reactions. The pooled ORs (95%CI) were 1.82 (0.93–3.53), 1.28 (0.47–3.46) and 2.32 (0.86–6.25), respectively (Fig. 3B-D).

### Subgroup analysis

We performed subgroup analysis according to ethnicity in order to investigate whether the association signal differs among different ethnic origin. In overall ADRs, BMT and hepatotoxicity subsets, studies were from Caucasian populations and Asian populations and subgroup analysis was performed. The results of subgroup analysis were also shown in **Fig. 2**. From the results, we can see that the pooled ORs (95%CI) of Caucasian population subgroup and Asian population subgroup in BMT subset were 6.97 (3.89–12.47) and 3.78 (0.72–19.85), respectively. This result still showed a significant association between *TPMT* polymorphisms and thiopurine-induce BMT in Caucasian populations while the association in Asian populations was not significant.In order to better investigate the association between *TPMT* heterosigosity and thiopurine-induced overall ADRs and BMT, an extra meta-analysis by excluding individuals with homozygosity genotypes of the genotyped *TPMT* polymorphisms was performed. The pooled ORs (95%CI) of overall ADRs and BMT subsets were 3.11 (1.64–5.88) and 5.56 (3.65–8.46), respectively. These results were consistent with the original results.

### Sensitivity analysis and publication bias

Sensitivity analysis was performed through sequential excluding individual studies. When the studies with small sample size [20, 22, 25, 30] were excluded in overall ADRs and BMT subsets, results remained consistent with original results. ORs (95%CI) in overall ADRs and BMT subsets were 3.48 (1.81–6.71) and 7.01 (4.53–10.85), respectively.

As a recommendation, tests for funnel plot asymmetry should not be used when there are fewer than 10 studies in the meta-analysis [33], thus only funnel plots of BMT subset is shown in **S1 Fig**. Egger’s test was used to provide statistical evidence of potential publication bias. The results did not suggest any evidence of publication bias except for the ‘pancreatitis’ subgroup (p = 0.027).

## Discussion

The thiopurine drug, 6-MP, and its pro-drug, AZA, have proven efficacy in IBD patients; therefore, these agents are prescribed to patients by physicians on a large scale [4]. However, safety concerns do exist, because moderate to serious adverse events may occur. Gastric intolerance, bone marrow toxicity, hepatotoxicity, pancreatitis, flu-like symptoms and skin reactions were among the most frequently reported clinically relevant adverse events. These events may be divided into dose-independent idiosyncratic reactions and dose-related, pharmacologically explainable toxicity [5]. In our study, we identified that there have been a number of variants tested in the identified studies for our meta-analysis. All the included studies identified the TPMT*3 family, while some studies tested additional variants. TPMT*3A is the most common variant allele in Caucasian populations, while TPMT*3C is the most common mutant allele in Asian and African populations [34, 35]. Genotyping for the TPMT*3 family of variant alleles (TPMT*3A, TPMT*3B and TPMT*3C) will detect over 92% of low activity alleles and inclusion of TPMT*2 pushes this to over 95% [15]. However, all the studies tested the most common polymorphisms and these would miss rare variants, which may lead to the underestimation of the effect of *TPMT* polymorphisms on thiopurine-induced ADRs. Data were insufficient to determine the optimum combination of *TPMT* alleles for testing.

The results of our meta-analysis demonstrated that patients who were *TPMT* polymorphism positive were at greater risk of overall ADRs (OR = 3.36, 95%CI: 1.82–6.19), which is consistent with previous studies [24, 26, 29, 31]. However, studies by Hindorf, U. [22] and by Zhu, Q. [30] indicated that there was no significant association between *TPMT* polymorphisms and overall ADRs. This may be due to a type-2 error, considering the small sample sizes in each study (54 patients and 52 patients, respectively). Results of sensitivity analysis showed that after excluding studies with small sample size, results remained consistent with the original results.

In our study, we found an association between *TPMT* polymorphisms and thiopurine-induced BMT (OR = 6.67, 95%CI: 3.88–11.47); this result is in agreement with previous studies, where it is well recognized that *TPMT* mutant patients are at greater risk for developing BMT. There is also no doubt in the literature that patients who are homozygous or compound heterozygous for a variant allele confer a very high-risk of early severe BMT. This was again confirmed by our study; as one was compound heterozygous [22] and 6 were homozygous [18, 20, 21, 24, 31] and were detected in all the 2,276 patients, all of the 7 petients experienced early, severe leucopenia requiring hospital management. After excluding individuals with homozygosity genotypes of the genotyped *TPMT* polymorphisms, pooled ORs (95%CI) in overall ADRs and BMT subsets were 3.11 (1.64–5.88) and 5.56 (3.65–8.46), respectively. These results were consistent with the original results, which indicated that *TPMT* heterosigosity was also associated with thiopurine-induced overall ADRs and BMT. Because of the life threatening nature of thiopurine-induced BMT, pretesting for *TPMT* genotype before the initiation of thiopurine therapy has increasingly been accepted clinically. Several guidelines recommend determining *TPMT* status before thiopurine therapy. However, these recommendations are considered to be premature from an evidence-based perspective, due to the absence of direct and crucial evidence that *TPMT* pretreatment testing decreases BMT-specific mortality [16]. Thus, white blood cell count analysis should be the mainstay for follow-up.

The association between *TPMT* polymorphisms and thiopurine-induced hepatotoxicity was not depicted in this meta-analysis (OR = 1.27, 95%CI: 0.60–2.71). *TPMT* polymorphisms were seldom detected in patients with thiopurine-induced hepatotoxicity, only in 6 of 79 patients. Increased levels of methylated inactive metabolite 6-methymercaptopurine (6-MMP) was introduced as a possible explanation for thiopurine-induced hepatotoxicity. Dubinsky et al.[36] have demonstrated that hepatotoxicity events in thiopurine-treated IBD patients have been associated with higher median 6-MMP levels (p < 0.05).

Our study revealed that *TPMT* polymorphisms were not associated with thiopurine-induced pancreatitis (OR = 0.97, 95%CI: 0.38–2.48). On the contrary, a recent study by Carvalho et al. reported a positive association between *TPMT* polymorphisms and the development of pancreatitis [17]. No definition of pancreatitis was given in this article; therefore we could not determine the reason for this difference. Pancreatitis is a dose-independent ADR, which seems to be independent of the accumulation of thiopurine metabolites and *TPMT* polymorphisms. Some researchers have suggested that a type 1 hypersensitivity reaction may be an explanation for dose-independent ADRs, including pancreatitis and gastric intolerance [37]. *TPMT* polymorphisms also failed to identify patients at risk for developing thiopurine-induced gastric intolerance, flu-like symptoms and skin reactions. Flu-like symptoms and skin reactions are also dose-independent ADRs, which may be not associated with *TPMT* polymorphisms, but are due to increased sensitivity to thiopurine adverse events[37].

There were several potential limitations in our study. Although we tried to analyze the association between *TPMT* polymorphisms and all the ADRs induced by thiopurine, the heterogeneous definitions of BMT, hepatotoxicity and pancreatitis are indeed problematic, indicating that the results of this meta-analysis should be interpreted with caution. Secondly, the included studies were performed in European and Asian countries. Further studies from different populations are needed because of the well-known ethnic differences in the *TPMT* allele’s distributions. Lastly, the development of thiopurine-induced ADRs is a multi-factorial event, caused by a co-influence of factors, other than variants in *TPMT* [28, 38], and a combined evaluation of the potential factors may enhance the correlation with ADRs. A study by Zalbala [38] reported variants associated with thiopurine-related BMT that was identified by a genome-wide association study (GWAS). They indentified that rs372996 in *interleukia 6 singnal transducer (IL6ST)* gene and re3749598 in *follistatin-like 5 (FSTL5)* gene as new bone marrow toxicity susceptibility candidate genes after thiopurine treatment in IBD patients. The ORs (95%CI) were 3.41 (1.71–6.78) and 3.67 (1.68–8.01), respectively.

Despite its limitations, our study does provide helpful insight into the potential association between *TPMT* polymorphisms and thiopurine-induced ADRs. The present meta-analysis demonstrates that *TPMT* polymorphisms strongly predicted overall ADRs and BMT in thiopurine-treated IBD patients, and this may translate to improved clinical outcome in the management of these patients. Our study also clearly indicates that thiopurine-induced hepatotoxicity, pancreatitis, gastric intolerance, flu-like symptoms and skin reactions are not associated with *TPMT* polymorphisms. In order to maximize efficacy, while minimizing the toxicity of thiopurine, several guidelines recommend *TPMT* testing in patients before commencing thiopurine treatment. Our findings may become powerful and indirect evidence for these recommendations in the absence of crucial, direct evidence. However, white blood cell count analysis should be the mainstay for follow-up.

## Materials and Methods

### Literature search strategy

Medline (using PubMed as the search engine), Web of science and Excerpta Medica Database (Embase) were searched to identify relevant publications published in English through July 22, 2014. Only human-related literature was searched. The following search words (in Tilte/Abstract fields) were used: ‘*TPMT*’ or ‘thiopurine S-methyltransferase’ or ‘thiopurine methytransferase’ **AND** ‘IBD’, or ‘inflammatory bowel disease’ or ‘ulcerative colitis’ or ‘Crohn’s disease’ **AND** ‘thiopurine’ or ‘azathioprine’ or ‘imuran’ or ‘6-mercaptopurine’ **AND** ‘adverse effects’ or ‘adverse reactions’ or ‘side effects’ or ‘adverse drug reactions’ or ‘toxicity’ or ‘toxicities’ or ‘adverse events’. We also performed a manual search of the references listed in the articles identified in the search for additional eligible studies. The search was conducted independently by two reviewers (YPL and HYW).

### Inclusion and exclusion criteria

The abstracts and full texts were read independently by the two reviewers (YPL and HYW). The following inclusion criteria were used: 1) studies that compared *TPMT* polymorphisms between with-ADRs and without-ADRs in IBD patients; 2) articles published in English and being human-related were included; 3) expert opinions supported by a preliminary literature review indicated that there was likely to be very few randomized, controlled trials (RCTs) on this topic; therefore, any study design (cross-sectional cohort, prospective cohort and case control studies) were included in this meta-analysis [39]; 4) all patients included in this meta-analysis were genotyped for *TPMT* polymorphisms. Studies on non-IBD patients were excluded. Reviews, letters, comments, and conference abstracts were also excluded because of limited data. Further, publications identified as duplicates were excluded.

### Data extraction strategy

Two reviewers (YPL and HYW) independently extracted relevant data from each eligible study. The following data were collected: author’s name, publication year, country, study type, number of enrolled patients, thiopurine dose, number of patients that were mutant-type *TPMT* with and without an ADR, *TPMT* polymorphism type, and number of homozygous mutant-type *TPMT*, allele frequencies and definitions of ADRs. Disagreements between reviewers were resolved by discussion or by consensus including a third author (QH).

### Statistical analysis

The meta-analysis was conducted using RevMan 5.3 software. Odds ratio (OR) with corresponding 95% confidence interval (CI) were calculated for the *TPMT* polymorphisms vs ‘overall ADRs’, ‘BMT’, ‘hepatotoxicity’, ‘pancreatitis’, ‘flu-like symptoms’, ‘gastric intolerance’, and ‘skin reactions’. Not all studies reported all ADRs analyzed in this meta-analysis, and so only studies that reported the adverse events of interest were analyzed for the association between *TPMT* polymorphisms and that adverse event. The included studies displayed heterogeneity concerning study designs, definitions of the ADRs, and the time to onset of thiopurine-induced ADRs. The degrees of included studies’ heterogeneity were explored using the chi-squared test of heterogeneity, and inconsistency index (I^2^). Considering the low statistical power of these tests, a p-value of <0.10 or an I^2^ >30% was defined as significant heterogeneity. ORs from different groups were combined using fixed or random effects models, which depends on the absence or presence of significant heterogeneity.

Sensitivity analysis was performed to assess the stability of the results; namely, a single study in the meta-analysis was deleted each time to reflect the influence of the individual data set to the overall OR. Publication bias was assessed by visual inspection of the funnel plot for symmetry, and formal statistical testing using the Egger test.

## Supporting Information

S1 Checklist PRISMA 2009 Checklist. (DOC)

S2 Checklist Genetic checklist. (DOCX)

S1 Fig Funnel plots of BMT subset meta-analysis. The dotted vertical line indicates the overall OR. S.E. = standard error, OR = odds ratio. Each circle represents an eligible study.
(EPS)

S1 Table Characteristics of 11 excluded studies. (DOCX)

S2 Table Characteristics of 14 included studies. (XLSX)
