# Supplementary material for: Association between Thiopurine S-methyltransferase Polymorphisms and Thiopurine-Induced Adverse Drug Reactions in Patients with Inflammatory Bowel Disease: A Meta-Analysis
Source: PLoS One. 2015 Mar 23;10(3):e0121745. doi: 10.1371/journal.pone.0121745 (PMC4370632; doi:10.1371/journal.pone.0121745)
Supplement: S1 Table — (DOCX) [file pone.0121745.s004.docx]

**S1 Table. Characteristics of the 11 excluded studies**

| **Author** | **Country** | **Study design** | **Participants** | ***TPMT* genotypes determined** | **Reasons for exclusion** |
| --- | --- | --- | --- | --- | --- |
| Gearry, R B[[1](#_ENREF_1)] | New Zealand; | Cross-sectional | 56 | *TPMT**2,*3A,*3C | Not all patients were genotyped |
| Lene O. Reuther [[2](#_ENREF_2)] | Denmark | Cross-sectional | 52 | *TPMT**2,*3A,*3B,*3C | Not related (Non-IBD patients) |
| Ban, Hiromitsu[[3](#_ENREF_3)] | Japan | Case-control | 111 | *TPMT**2,*3A,*3B,*3C | Not related (Dose of thiopurine not mentioned) |
| Noritaka Takatsu[[4](#_ENREF_4)] | Japan | Cross-sectional | 147 | *TPMT**2,*3A,*3B,*3C, *8 | Incomplete data |
| Uchiyama, K[[5](#_ENREF_5)] | Japan | Cross-sectional | 16 | *TPMT**2,*3A,*3B,*3C,,*3D,*4,*5,*6,*7,*8 | Not related (Incomparable control group) |
| Cao, Q[[6](#_ENREF_6)] | China | Cross-sectional | 189 | *TPMT**3A,*3B,*3C | Incomplete data |
| Dewit, O[[7](#_ENREF_7)] | Belgian | Cross-sectional | 61 | Sequencing | Not all patients were genotyped |
| Becquemont, L[[8](#_ENREF_8)] |  |  |  |  | Not related (similar to conference abstract) |
| Newman, W G[[9](#_ENREF_9)] | UK | Randomized controlled trial | 333 | *TPMT**2,*3A,*3B,*3C | Not related (assessment of whether *TPMT* genotyping prior to azathioprine reduces ADRs) |
| Fangbin, Z[[10](#_ENREF_10)] | China | Case-control | 499 | *TPMT**2,*3A,*3B,*3C | Not related (not about association between *TPMT* polymorphism and thiopurine-induced ADRs) |
| Carvalho, A T P[[11](#_ENREF_11)] | Brazil | Cross-sectional | 219 | *TPMT**2，*3A, *3C | Incomplete data |

1. Gearry RB, Barclay ML, Burt MJ, Collett JA, Chapman BA, et al. (2003) Thiopurine S-methyltransferase (*TPMT*) genotype does not predict adverse drug reactions to thiopurine drugs in patients with inflammatory bowel disease. Aliment Pharmacol Ther 18: 395-400.

2. Reuther LO, Vainer B, Sonne J, Larsen NE (2004) Thiopurine methyltransferase (*TPMT*) genotype distribution in azathioprine-tolerant and -intolerant patients with various disorders. The impact of *TPMT* genotyping in predicting toxicity. Eur J Clin Pharmacol 59: 797-801.

3. Ban H, Andoh A, Tanaka A, Tsujikawa T, Sasaki M, et al. (2008) Analysis of Thiopurine S-Methyltransferase Genotypes in Japanese Patients with Inflammatory Bowel Disease. Intern Med 47: 1645-1648.

4. Takatsu N, Matsui T, Murakami Y, Ishihara H, Hisabe T, et al. (2009) Adverse reactions to azathioprine cannot be predicted by thiopurine S-methyltransferase genotype in Japanese patients with inflammatory bowel disease. Journal of Gastroenterology and Hepatology (Australia) 24: 1258-1264.

5. Uchiyama K, Nakamura M, Kubota T, Yamane T, Fujise K, et al. (2009) Thiopurine S-methyltransferase and inosine triphosphate pyrophosphohydrolase genes in Japanese patients with inflammatory bowel disease in whom adverse drug reactions were induced by azathioprine/6-mercaptopurine treatment. J Gastroenterol 44: 197-203.

6. Cao Q, Zhu Q, Shang Y, Gao M, Si J (2009) Thiopurine Methyltransferase Gene Polymorphisms in Chinese Patients with Inflammatory Bowel Disease. Digestion 79: 58-63.

7. Dewit O, Moreels T, Baert F, Peeters H, Reenaers C, et al. (2011) Limitations of extensive *TPMT* genotyping in the management of azathioprine-induced myelosuppression in IBD patients. Clin Biochem 44: 1062-1066.

8. Becquemont L, Alfirevic A, Amstutz U, Brauch H, Jacqz-Aigrain E, et al. (2011) Practical recommendations for pharmacogenomics-based prescription: 2010 ESF-UB Conference on Pharmacogenetics and Pharmacogenomics. Pharmacogenomics 12: 113-124.

9. Newman WG, Payne K, Tricker K, Roberts SA, Fargher E, et al. (2011) A pragmatic randomized controlled trial of thiopurine methyltransferase genotyping prior to azathioprine treatment: The TARGET study. Pharmacogenomics 12: 815-826.

10. Fangbin Z, Xiang G, Minhu C, Liang D, Feng X, et al. (2012) Should thiopurine methyltransferase genotypes and phenotypes be measured before thiopurine therapy in patients with inflammatory bowel disease? Ther Drug Monit 34: 695-701.

11. Carvalho ATP, Esberard BC, Froes RSB, Rapozo DCM, Grinman AB, et al. (2014) Thiopurine-methyltransferase variants in inflammatory bowel disease: Prevalence and toxicity in Brazilian patients. World J Gastroenterol 20: 3327-3334.
